# A preliminary investigation of yoga as an intervention approach for improving long-term weight loss: A randomized trial

**DOI:** 10.1371/journal.pone.0263405

**Published:** 2022-02-04

**Authors:** Jessica L. Unick, Shira I. Dunsiger, Beth C. Bock, Sally A. Sherman, Tosca D. Braun, Rena R. Wing

**Affiliations:** 1 Weight Control and Diabetes Research Center, The Miriam Hospital, Providence, Rhode Island, United States of America; 2 Department of Psychiatry and Human Behavior, Warren Alpert Medical School at Brown University, Providence, Rhode Island, United States of America; 3 Department of Behavioral and Social Sciences, Brown University School of Public Health, Providence, Rhode Island, United States of America; 4 Centers for Behavioral and Preventive Medicine, The Miriam Hospital, Providence, Rhode Island, United States of America; 5 School of Education, University of Pittsburgh, Pittsburgh, Pennsylvania, United States of America; Pennington Biomedical Research Center, UNITED STATES

## Abstract

**Objective:**

Yoga targets psychological processes which may be important for long-term weight loss (WL). This study is the first to examine the feasibility, acceptability, and preliminary efficacy of yoga within a weight management program following WL treatment.

**Methods:**

60 women with overweight or obesity (34.3±3.9 kg/m^2^, 48.1±10.1 years) were randomized to receive a 12-week yoga intervention (2x/week; YOGA) or a structurally equivalent control (cooking/nutrition classes; CON), following a 3-month behavioral WL program. Feasibility (attendance, adherence, retention) and acceptability (program satisfaction ratings) were assessed. Treatment groups were compared on weight change, mindfulness, distress tolerance, stress, affect, and self-compassion at 6 months. Initial WL (3-mo WL) was evaluated as a potential moderator.

**Results:**

Attendance, retention, and program satisfaction ratings of yoga were high. Treatment groups did not differ on WL or psychological constructs (with exception of one mindfulness subscale) at 6 months. However, among those with high initial WL (≥5%), YOGA lost significantly more weight (-9.0kg vs. -6.7kg) at 6 months and resulted in greater distress tolerance, mindfulness, and self-compassion and lower negative affect, compared to CON.

**Conclusions:**

Study findings provide preliminary support for yoga as a potential strategy for improving long-term WL among those losing ≥5% in standard behavioral treatment.

## Introduction

Nearly 70% of adults in the United States are living with overweight or obesity [1]. Standard behavioral weight loss (SBWL) interventions effectively reduce body weight, but rates of long-term weight loss (WL) success are less than optimal [2, 3]. Unsuccessful long-term WL may result from both physiological adaptations favoring weight regain, and poor long-term behavioral adherence to dietary and physical activity regimens [4, 5]. Previous findings indicate that successful WL maintainers have an improved ability to cope with life stressors, negative mood states, and physiological and hedonic urges to eat [6–8], while weight regainers exhibit affective or distress intolerance and eat to regulate aversive mood states [6, 9]. Thus, while it would seem appropriate that interventions target these psychological factors to improve long-term WL following an initial WL period, these mechanisms have received limited attention as an intervention target.

One potential approach for targeting factors associated with poor long-term WL success is yoga. Yoga is a mind-body intervention shown to improve physical and psychological well-being [10, 11], and is an effective treatment for numerous chronic conditions [12–14]. Moreover, yoga offers promise for strengthening the cognitive skills needed for maintaining important weight-related behaviors long-term. Specifically, studies show that yoga reduces stress [15] and improves mood [16, 17], self-efficacy [18], mindfulness [19, 20], self-compassion [21, 22], and distress tolerance [23, 24]. Despite these psychological benefits, yoga has not been examined as a potential intervention approach for improving long-term WL outcomes. Many styles of yoga have been shown to elicit a lower energy expenditure than aerobic exercise [25], thus it is hypothesized that yoga works via improvements in these psychological factors, leading to better adherence to diet and exercise prescriptions in the face of negative emotions, stress, or other aversive mood states. For example, preliminary data indicate a favorable effect of yoga on weight among individuals with overweight/obesity [26], and also on binge eating, emotional eating, mindful eating, and dietary choices [27–29].

The physical and cognitive skills practiced within yoga, which target multiple underlying psychological processes (e.g., stress, mindfulness, distress tolerance), more closely align with long-term WL or WL maintenance skills (e.g., relapse prevention, coping strategies) rather than important behaviors necessary for initial WL (e.g., self-monitoring diet and exercise). While previous reviews have assessed the effect of yoga on weight [26, 30], prior studies are plagued by small samples sizes of normal weight individuals, few randomized trials, and limited use of yoga in conjunction with a dietary or WL intervention. Further, yoga has not been considered as a strategy for improving *long-term* WL. The current study addresses these limitations and is an important first step towards investigating yoga as an approach for improving WL following standard behavioral treatment.

The purpose of this randomized trial is to evaluate the feasibility, acceptability, and preliminary efficacy of a 12-week yoga intervention, following a 3-month WL program, among women with overweight/obesity. Yoga participants are compared to a structurally equivalent control condition on changes in weight, psychological constructs (e.g., stress, mindfulness, distress tolerance), and aerobic exercise minutes, to examine whether the practice of yoga reduces participation in moderate-intensity exercise.

## Materials and methods

### Recruitment

**Fig 1:**
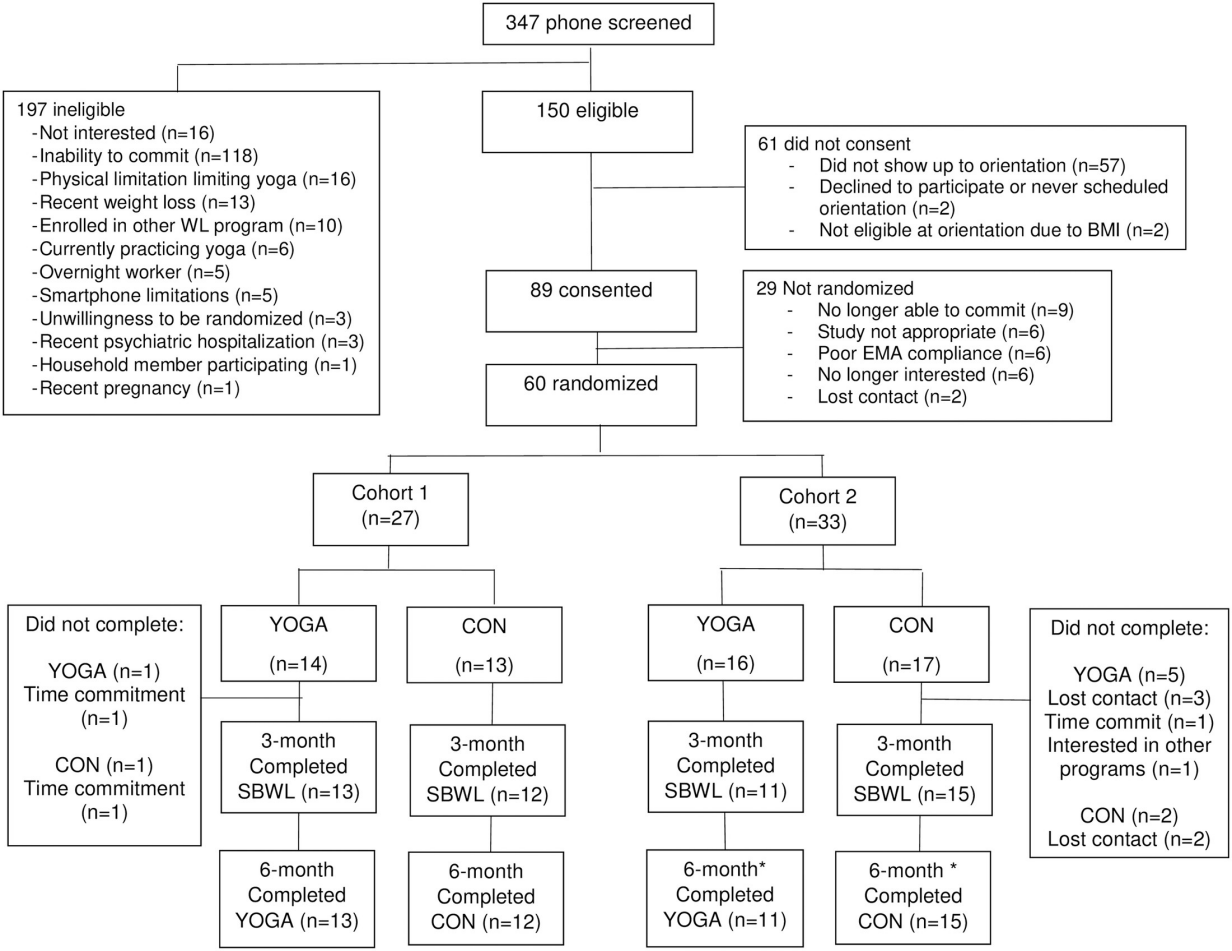
Consort diagram. * While all participants completed the 6-month assessment visit, 1 YOGA and 2 CON participants from Cohort 2 did not continue to participate in the intervention beyond week 6 due to childcare issues as a result of the COVID-19 pandemic.

Participants were primarily recruited via generic WL advertisements on Facebook (e.g., enrolling now for a new weight loss study which examines approaches for improving long-term weight loss), as we were recruiting for other WL studies simultaneously; thus these advertisements did not specifically mention yoga. Interested individuals were invited to complete an initial online screener which screened for BMI, age, and availability on Tuesday and Thursday evenings (when the intervention would be delivered). Those meeting these criteria and providing their contact information were called by a member of the research team to complete a phone screen. During this phone screen, the study was described in detail (this was when participants first learned that it included yoga) and individuals were asked questions to assess their eligibility and interest in the study. The number of individuals completing the phone screen who were eligible and ineligible are shown in Fig 1. Eligible participants were invited to an orientation session where they could find out more information about the study and sign informed consent if interested.

### Participants

Sixty women with overweight or obesity (BMI: 25 to <40.0 kg/m^2^), aged 18 and 60, participated in this study between January 2019 and December 2020. Exclusionary criteria included recent WL (≥10 pounds during past 6 months), current or recent enrollment in a WL program, plans to become pregnant in the next year, any condition that would limit ability to exercise (i.e., orthopedic limitations), serious psychiatric disorders (i.e., involving a hospitalization within the past 2 years), engagement in stress-management or mindfulness-based treatment (e.g., yoga, Tai Chi) within the past year, or any impairments limiting the use of a smartphone device. Men were excluded given that hedonic eating is more prevalent in women [31].

### Overall study design

Participants were recruited in two cohorts and received a 3-month standard-behavioral weight loss (SBWL) program which included weekly group sessions (1x/week) and diet and exercise goals. Following the completion of this program, participants received either 12 weeks of Iyengar yoga (YOGA; 2x/week) or 12 weeks of culinary and nutrition education classes (2x/week), which served as the control (CON) condition. While participants were randomized at baseline (using a treatment allocation sequence generated using a random numbers table) so that they could remain with the same cohort of individuals throughout the entire intervention, they were not informed of their randomization assignment until the final week of SBWL treatment. Assessments were completed at baseline, post-SBWL (3 months), and upon completion of the yoga or culinary/nutrition classes (6 months). Written informed consent was obtained prior to enrollment, all study procedures were approved by The Miriam Hospital’s Institutional Review Board, and this study was registered on ClinicalTrials.gov (NCT03799289).

### Sample size calculations

The study was powered for a total sample of N = 60 participants and was based on the budgetary and time constraints associated with an R03 grant. Given a sample of size n = 60 (30/group) and a two-sided alpha of .05, we estimated that we would have 80% power to detect a medium effect size (Cohen’s d ≥0.51) for the between-group differences at 6 months, assuming a pre-post correlation of at least 0.71. This was consistent with previous studies, which reported large effect sizes (Cohen’s d ≥1.0) for the effect of yoga on perceived stress [32], distress tolerance [24], and mindfulness [19]. Further, the stage model of behavioral therapy research [33] recommended 15–30 participants per group for a stage 1b pilot study; thus our sample size was consistent with those recommendations. Although the study was not originally powered for the analytic methods described in this paper, a post-hoc power calculation would suggest that given a sample of size n = 60, and a distribution of initial weight loss (as described below), we would have had 71% power to detect medium-sized conditional (moderator) effects on median weight loss at 6 months.

### Standard behavioral weight loss program (common to both treatment groups)

The 3-month, in-person SBWL program was designed to produce a 1–2 lb WL per week and was modeled after the Look AHEAD Trial [34]. Participants attended weekly, group-based classes at our research center (12 classes in total), which were 60 minutes in duration and lead by two interventionists, one a PhD level clinical psychologist and one with a bachelor’s degree in nutrition. They were given a calorie intake goal of 1200–1800 kcals/day and were given a moderate-intensity aerobic exercise prescription (progressed from 75 to 200 min/week). Group sessions began with a brief check-in related to successes and barriers from the previous week, which was followed by lecture and discussion related to the weekly lesson (printed copy provided to participants). Intervention content focused on key behavioral strategies for modifying diet and exercise behaviors (e.g., stimulus control, problem solving, goal setting) and participants were instructed to record food intake, weight, and exercise daily, using written or electronic diaries which were submitted weekly to research staff. Written feedback was provided weekly during weeks 1–4 and monthly during weeks 5–12. Following completion of SBWL treatment, participants were encouraged to continue self-monitoring and daily weighing; however, diaries were not submitted and yoga and culinary/nutrition instructors did not discuss these weight-control practices.

### Yoga intervention

YOGA participants received a 12-week, group-based Iyengar yoga intervention following the 12-week SBWL program. Classes were held twice per week for 60 minutes at the Weight Control and Diabetes Research Center (same location as SBWL treatment). Iyengar yoga [35] is a form of hatha yoga, which incorporates breathing, postural, and meditation practices and utilization of ‘props’ (e.g., straps, blocks, blankets, chairs), to assist individuals in maintaining the proper alignment and to reduce the risk of injury. Compared to Vinyasa, Iyengar yoga has a lower energy expenditure and was selected to avoid a potential confound with assessments of aerobic physical activity. Group classes were led by two certified Iyengar yoga instructors (13–15 years of teaching experience) and consisted of a brief warm-up (~5–7 minutes), a period of more intense poses (~35 minutes), a cool-down consisting of more relaxing poses (~3–7 minutes), breath work (~7–10 minutes), and Savasana (~2–10 minutes). Yoga instructors were informed of the basic hypotheses of the research project, but were not active members of the research team. Further, as shown in Table 3, participants were encouraged to engage in self-initiated yoga practice at home; they were provided with resources for practicing yoga at home, and given weekly notecards, helping them to apply the learned yoga skills to weight-control behaviors.

#### Differences in yoga intervention by cohort

There were several differences between Cohorts 1 and 2 due to the COVID-19 pandemic and feedback received from participants following Cohort 1 (see Table 1). Specifically, Cohort 1 group classes were delivered in-person, while Cohort 2 classes started in-person (weeks 1–3) and transitioned to virtual classes (i.e., live classes with participants using video cameras for the final 9 weeks) due to COVID-19. Further, as a result of informal conversations with yoga participants from Cohort 1 and examination of self-initiated yoga practice and program satisfaction data, changes were made to the yoga intervention for Cohort 2 to promote additional self-initiated yoga practice, help participants make the connection between their yoga practice and weight-control behaviors, and increase the level of difficulty of yoga sessions, as participants verbally indicated that classes were not sufficiently challenging. Moreover, as assessed by the Essential Properties of Yoga Questionnaire [36], the average number of yoga postures per class did not differ by cohort, but on average, Cohort 2 held individual poses for a longer duration and had a shorter Savasana, compared to Cohort 1.

**Table 1:** Comparison of yoga Intervention by cohort.

	Cohort 1	Cohort 2
Yoga		
Format of classes	All yoga sessions were group-based and in-person	Group-based in-person yoga classes until week 3, followed by a 2-week pause (participants encouraged to use 60-minute yoga video, filmed by instructors, on their own during scheduled group time). Following 2-week pause, group classes resumed live, in a virtual format for the remaining 9 weeks*
Frequency of classes	Twice per week for 12 weeks	Twice per week for 12 weeks
Average number of yoga postures per class	30–39 postures (note: every repetition of the same posture was included in the count)	30–39 postures (note: every repetition of the same posture was included in the count)
Average duration of individual poses	36.0±7.9 seconds	44.6±2.0 seconds (diff between cohorts: p<0.001)
Average length of Savasana	8.9±1.4 minutes	4.3±2.8 minutes (diff between cohorts: p<0.001)
Home-based (self-initiated) yoga practice prescription	‘Loose’ recommendation for home-based yoga practice (i.e., participants encouraged to practice yoga on their own throughout week, but no specific prescription was provided)	Participants were provided with formal home-based yoga prescription each week. This prescription was reviewed at the beginning of each class by instructor: Weeks 1–2: 2 days/wk for 10 min Weeks 3–5: 2 days/wk for 15 min Weeks 6–9: 3 days/wk for 15 min Weeks 10–12: 3 days/wk for 20 min
Strategies for promoting self-initiated yoga practice	Participants provided with home-practice sequence handouts every few weeks. Each handout provided 7–8 poses with instructions to hold poses for 5–8 breaths and repeat two times.	Participants were provided with 10- and 15-min audio recordings to guide self-initiated yoga practice and asked to complete a weekly homework worksheet (worksheet components: self-report of daily yoga min practiced and answer weekly question reflecting upon their yoga experiences). Instructors reviewed the homework assignment weekly during class. Completed homework logs were sent to research staff weekly, but no feedback was provided.
Method for connecting yoga practice to weight-related behaviors	Weight-related behaviors not discussed in class. Participants were provided with weekly notecards to help them apply a specific yoga principle from class (e.g., noticing thoughts, determining whether thoughts are helpful or unhelpful, balancing effort and ease, being self-compassionate, contentment, focus on breath, etc) to their weight control practices. Notecards were handed out at the end of class and participants were encouraged to read them on their own.^†^	Weight-related behaviors not discussed in class. Participants were provided with weekly notecards to help them apply a specific yoga principle to their weight control practices. Yoga instructors read the notecard aloud at the beginning of each class in case participant did not take the time to read it on their own.

* Modification due to the COVID-19 pandemic.

^†^The decision was made not to discuss any weight-related behaviors during yoga class, but rather to provide weekly notecards to supplement class content to order to stay true to traditional Iyengar yoga for translation purposes.

### Culinary & nutrition education intervention

Participants randomized to CON received 12 weeks of culinary/nutrition education classes (2x/week, 60 minutes/session), led by dieticians or culinary experts, following the 3-month SBWL program. This group served as a control condition; weight loss was never discussed and no behavioral strategies for changing diet were used. Instead, lessons focused on educational content related to nutrition and cooking, and healthy recipes were provided and discussed. Example topics included proper knife skills when cooking, washing produce to remove pesticides, different types of whole grains and fats, fluid, fiber, and sodium recommendations, and information on vitamins, minerals, and phytochemicals. This was selected as the control condition as the contact hours were equivalent to YOGA, and intervention content was relevant to WL (e.g., focused on diet and cooking) to promote attendance, but would not likely influence weight or psychological constructs targeted by yoga (e.g., mindfulness, stress, distress tolerance). Due to the COVID-19 pandemic, the method of intervention delivery varied by cohort (i.e, Cohort 1: all in-person classes, Cohort 2: 3 weeks in-person and 9 weeks virtual), but did not differ in content.

### Measures

In-person assessments were completed at baseline, 3 months (following SBWL), and 6 months (following yoga or cooking/nutrition intervention).

#### Weight and height

Height was measured (baseline only) to the nearest millimeter using a stadiometer, and weight was measured at all assessments to the nearest 0.1 kg using a digital scale.

#### Objective assessment of physical activity

Participants wore a Sensewear armband (Body Media Inc.) for 10 consecutive days at each assessment period. This device provides valid and reliable estimates of exercise intensity and energy expenditure [37]. Moderate-to-vigorous intensity physical activity (MVPA; ≥10 minutes and ≥3 METs) was calculated. To be included in the analyses, participants needed ≥4 ‘valid’ days (i.e., armband wear time ≥8 hours/day) at any given assessment period.

#### Questionnaire measures

At each assessment, participants completed a series of questionnaires related to psychological constructs targeted by the yoga intervention. *Perceived stress* was measured via the 10-item Perceived Stress Scale [38] which assesses the degree to which situations in one’s life are appraised as stressful, with a total score computed. *Dispositional mindfulness* was assessed via the 39-item Five Facet Mindfulness Questionnaire [39] which has five subscales related to observing, describing, being non-judgmental and non-reactive to feelings, thoughts, and emotions, and acting with awareness in the moment. *Distress tolerance*, or the capacity to experience and withstand negative psychological states, was assessed using the 15-item Distress Tolerance Scale [40] which provides a total score. *Positive and negative affect* were assessed via the 20-item Positive and Negative Affect Schedule [41] which asks participants to rate different feelings and emotions over the previous week and has 2 subscales (positive and negative affect). *Self-compassion* was assessed using the 26-item Self-Compassion Scale [42] which measures how often people respond to feelings of inadequacy or suffering with self-kindness, self-judgment, common humanity, isolation, mindfulness, or over-identification (7 subscales and total score).

#### Feasibility and acceptability of the yoga intervention

Feasibility was assessed by 6-month retention rates (i.e., completion of assessment visit) and adherence to the yoga intervention (i.e., attendance at yoga classes, retrospective recall of self-initiated yoga practice, and weekly self-reported yoga practice from homework logs (Cohort 2 only). Acceptability was assessed via questionnaire which queried participants on their level of enjoyment and satisfaction with the program (see Table 2 for list of questions).

**Table 2:** Program satisfaction scores for YOGA participants.

	Overall (n = 24)	Cohort 1 (n = 13)	Cohort 2 (n = 11)	p-value for difference between cohorts
How satisfied were you with the yoga program you received over the past 3 months? (1 = very dissatisfied, 10 = very satisfied)	7.6±2.9	7.85±2.7	7.36±3.2	0.70
How satisfied were you that you were randomized to the yoga condition? (1 = very dissatisfied, 10 = very satisfied)	7.8±3.4	7.7±3.4	7.8±3.6	0.93
How much did you enjoy the yoga program over the past 3 months? (1 = did not enjoy at all, 10 = enjoyed very much)	7.6±2.9	7.8±2.7	7.5±3.3	0.80
How much do you believe that your participation in the yoga classes helped you to control your weight? (1 = did not help at all, 7 = very helpful)	5.0±1.9	5.3±1.7	4.7±2.1	0.46
How would you rate the quality of the yoga teaching you received?*	4.6±0.7	4.7±0.5	4.5±0.9	0.43
How would you rate the competency of your instructors?*	4.7±0.7	4.9±0.3	4.5±0.9	0.10
How well did your yoga instructors present concepts and techniques?*	4.6±0.7	4.9±0.4	4.4±0.9	0.10

*response options: 1 = poor, 2 = below average, 3 = average, 4 = above average, 5 = outstanding.

#### Statistical analysis

Baseline demographics, retention, adherence, and program satisfaction were summarized and compared between treatment arms and/or cohorts, using t-tests for continuous variables and chi-squared tests for categorical variables. Graphical methods were used to examine the distribution of outcome variables prior to analysis.

Using a quantile regression model, we examined treatment effects on WL from 3 to 6 months, controlling for initial WL (baseline to 3 months), treatment group, cohort, and treatment x cohort. Quantile regression models regress median outcomes on predictors instead of the mean outcomes, which is optimal in situations where the outcome is not normally distributed. In this case, weight change was skewed and transformations did not adequately bring the outcomes towards normality. As a subsequent step, we used a similar analytic approach to examine whether treatment effects were moderated by initial WL (defined as a 3-month WL <5% or ≥5%).

To examine potential treatment effects on psychological variables, we used a series of longitudinal mixed effects models which regressed outcomes at 3 and 6 months on baseline value, time, treatment x time, cohort, and treatment x cohort. A subsequent model examined whether treatment effects were moderated by initial WL. A similar analytic strategy was used to examine treatment effects on MVPA outcomes (excluding yoga practice as part of the study).

Models were estimated using likelihood/quasi-likelihood approaches, making use of all available data without directly imputing missing outcomes. Likelihood and quasi-likelihood approaches to estimation provide consistent estimates of regression parameters. Analysis was run in SAS 9.3 and significance level set at .05 a priori.

## Results

### Participants

Randomized participants (n = 60) had a mean BMI of 34.3±3.9 kg/m^2^, were 48.1±10.1 years of age, and predominately White (83.3%) and non-Hispanic (90%). As shown in Table 3, YOGA (n = 30) and CON (n = 30) did not differ on baseline demographic variables. Cohort 1 (n = 27) had a higher baseline BMI (35.4±3.2 kg/m^2^) compared to Cohort 2 (n = 33; 33.4±4.2 kg/m^2^, p = 0.048)—there were no other baseline differences by cohort.

**Table 3:** Baseline characteristics by intervention arm.

	YOGA (n = 30)	CON (n = 30)	p-value for difference between groups
Age (years)	48.2±10.1	48.0±10.2	0.95
Height (cm)	161.9±5.7	162.8±6.7	0.59
Weight (kg)	89.4±12.9	90.6±11.0	0.71
BMI (kg/m^2^)	34.0±4.0	34.5±3.8	0.67
% White (n/%)	25 (83.3%)	25 (83.3%)	1.00
% Hispanic (n/%)	4 (13.3%)	2 (6.7%)	0.39
MVPA (min/wk)	92.2±109.1	84.1±89.9	0.76

### Retention and intervention adherence

Of the 60 participants randomized, 51 (85%) completed the SBWL program and 3-month assessment visit and thus were informed of their randomization assignment. Reasons for dropout are shown in Fig 1. Of those who received yoga (n = 24) or cooking/nutrition classes (n = 27), 100% completed the 6-month assessment. However, despite all participants completing this assessment, three Cohort 2 participants (n = 1 YOGA and n = 2 CON) could no longer participate in the intervention classes due to not having childcare once COVID-19 shutdowns began.

Averaged across cohorts, participants in YOGA and CON attended a similar number of yoga (75.4±24.6%) or nutrition classes (75.9±27.1%, p = 0.94). Interestingly, Cohort 1 YOGA participants attended 69.6±21.9% of classes, whereas Cohort 2, in which most classes were delivered virtually, had 82.2±26.8% attendance (p = 0.22). This percentage increased to 89.6±11.5% in Cohort 2 when the one participant, who was unable to attend any virtual classes due to COVID-19, was removed from the analyses (difference between cohorts: p = 0.01).

### Self-initiated yoga practice

Yoga practice outside of group classes was assessed via retrospective questionnaire (both cohorts) and weekly homework logs (Cohort 2 only). Over the 3-month yoga intervention period, self-initiated yoga practice (days which yoga was practiced ≥5 minutes, excluding group classes) differed by cohort. In Cohort 1, only 15.4% of participants reported engaging in ≥2 days/week of self-initiated yoga practice, compared to 72.7% in Cohort 2. Similarly, 69.2% reported practicing on their own never or less than 1 day/week in Cohort 1, whereas only 18.2% of Cohort 2 participants fell into these categories.

Homework log data from Cohort 2 indicates that participants submitted 77.9±28.8% of homework logs (85.7±13.4% after removing one participant who could no longer participate due to COVID-19 related childcare issues). Of the 10 Cohort 2 participants who attended yoga classes virtually, mean weekly yoga practice was 163.7±42.6 min/week (this includes group-based yoga practice, and only includes weeks when logs were submitted: 10.3±1.6 weeks), indicating that on average participants engaged in >40 minutes/week of self-initiated yoga practice.

### Program satisfaction

When participants rated how satisfied they were with the yoga or cooking/nutrition program over the previous 3 months, there was no difference between YOGA (7.6±2.9 on 1–10 scale, a higher number indicating more satisfaction) and CON participants (8.3±2.4, p = 0.40). Table 3 presents additional measures of program satisfaction among YOGA participants, stratifying by cohort. Overall, participants enjoyed the yoga intervention, were highly satisfied with the program, and were glad they were randomized to the YOGA condition. There were no differences between cohorts.

### Weight

In the aggregate sample (N = 51), participants decreased their median weight (kg) from 91.7kg (IQR: 84.9–98.2) at baseline to 85.3kg (IQR: 76.2–92.9) at 3 months and 85.6kg (IQR:76.5–93.6) at 6 months. Adjusted models did not suggest any significant differences between conditions in median WL from 3 to 6 months, controlling for baseline (p = .93). However, participants randomized to YOGA lost less weight during SBWL treatment (3 months: -5.2±3.3%) compared to CON (-7.6±3.4%). Further, there were no significant differences in weight loss by cohort (p’s>.06).

**Fig 2:**
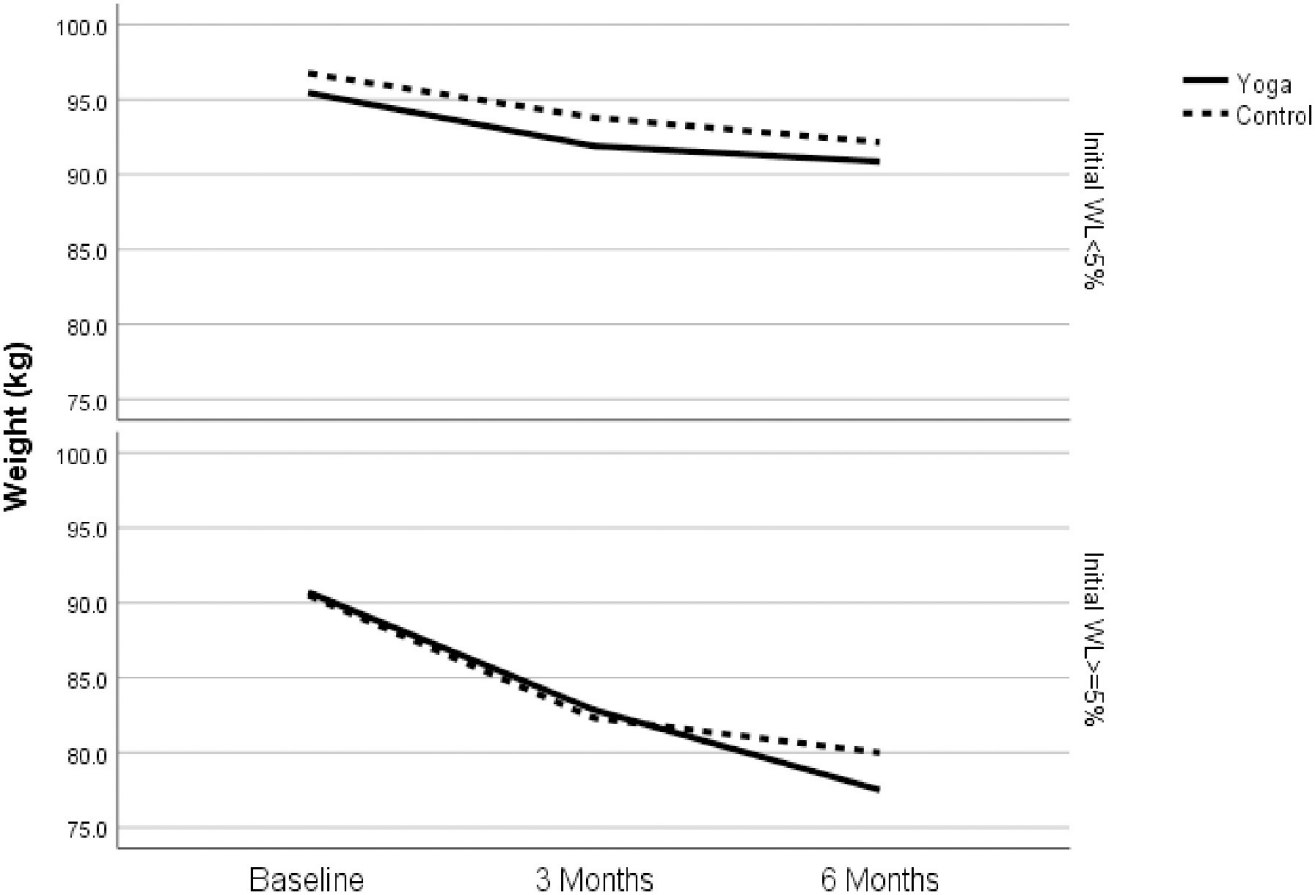
Adjusted median weight change over time by treatment arm and initial weight loss category. Initial weight loss is defined as percent weight loss achieved during behavioral weight loss treatment (3-month weight loss).

Given these differences in 3-month WL (i.e., initial WL) by treatment arm, it was considered as a moderator of intervention effects on 6-month weight change. Among those with *high* initial WL (3-month WL ≥5%) significant differences favoring YOGA in median WL between 3 and 6 months were observed, when controlling for initial WL and cohort (Fig 2). Specifically, among those with *high* initial WL, median 6-month WL (from baseline) for YOGA was -9.00 kg (5.15–9.57) which was significantly greater than CON (-6.65 (0.45–7.5). This corresponds to a difference in median percent WL of 3.5% between conditions (95% CI:.005-.139). There were no between-group differences in WL from 3–6 months among those with *low* initial WL (3-month WL <5%). Compared to individuals with *low* initial WL, those with *high* initial WL attended more yoga classes (85.8% vs. 61.8%, p = .01), but there were no differences between initial WL groups in cooking class attendance (77.2% vs. 64.6%, p = .31).

### Psychological constructs

At 6 months, compared to CON, YOGA had higher scores on the non-reacting subscale of the mindfulness questionnaire (b = 2.51, SE = 1.15, p = .04) with no other significant between-group differences in psychological constructs over time (p’s>.11) However, as shown in Table 4, the effect of the YOGA on these psychological constructs at 6-months varied as a function of initial WL (assessed at 3 months). Among those with *high* initial WL, relative to CON, YOGA resulted in greater distress tolerance, mindfulness (observing, non-judging, and non-reacting subscales and total score), and self-compassion (self-kindness, and total scores), as well as lower negative affect and self-compassion (isolation, judgement subscales; lower scores on these subscales are indicative of more self-compassion). Among those with *low* initial WL, YOGA had higher negative affect and lower positive affect compared to CON.

**Table 4:** Between-group differences in psychological constructs at 6 months stratified by initial weight loss.

	Low Initial WL(<5%)	High Initial WL (≥5%)
**Distress Tolerance (DTS)**	0.12(.31)	0.29(.02)*
**Five Facet Mindfulness**		
**(FFMQ)** ⱡ		
**Observing**	-1.68(2.63)	4.57(1.29)*
**Describing**	-0.94(2.78)	2.10(1.96)
**Non-Judging**	-0.60(2.49)	3.53(1.79)*
**Non-Reacting**	1.30(2.22)	3.96(1.43)*
**Acting with Awareness**	-2.28(1.51)	1.85(1.46)
**Total score**	-4.78(7.94)	16.23(5.26)*
**PANAS**		
**Positive**	-9.78(3.14)*	1.82(2.66)
**Negative**	7.44(3.45)*	-1.82(0.27)*
**Perceived Stress**	4.96(3.37)	-2.35(2.19)
**Self-Compassion** ^π^		
**Self-kindness**	-.38(.31)	.48(.21)*
**Self-judgment**	-.15(.30)	-.69(.20)*
**Common humanity**	-.59(.43)	31(.26)
**Isolation**	.08(.40)	-.50(.25)*
**Mindfulness**	-.17(.42)	.25(.21)
**Over-identified**	-.42(.40)	-.44(.30)
**Total Score**	-.17(.28)	46(.19)*

Regression coefficients (standard errors) are presented and represent differences in mean scores between YOGA and CON at 6 months adjusting for earlier time points. A b>0 suggests higher scores at 6 months among YOGA participants relative to CON and a b<0 suggests lower scores at 6 months among YOGA participants.

*p < .05 for between-group differences.

^ⱡ^FFMQ subscales: observing (noticing or attending to internal feelings and thoughts about external simulation), describing (labeling feelings, thoughts and experiences with words), non-judging of inner experience (taking a non-evaluative stance toward internal thoughts and feelings), non-reactivity to inner experience (allowing emotions and thoughts to come and go, without being interfered by them), and acting with awareness (attending to what is happening in the present). Total score is a sum of all subscales.

^Π^ Self-compassion subscales: self-kindness (trying to be loving toward oneself when feeling emotional pain), self-judgment (being disapproving and judgmental about personal flaws and inadequacies), common humanity (trying to see one’s failings as part of the human condition), isolation (when think about inadequacies it produces feelings of being separated and cut off from the rest of the world), mindfulness (trying to take a balanced view of the situation when something painful happens), and over-identification (obsessing and fixating on everything that’s wrong when feeling down). Note: higher scores for self-judgment, isolation, and over-identification indicate less self-compassion.

### Aerobic physical activity

Given the concern that yoga could reduce participation in aerobic exercise (i.e., yoga performed *in place of* aerobic exercise), MVPA was assessed. Overall, participants were highly compliant to wearing the armband (9.6±1.1 ‘valid’ days, 14.1±1.5 hours/day). Regression models did not suggest significant between-group differences in weekly minutes of MVPA accumulated in bouts ≥10 minutes (p = .91). Additionally, there were no differences in treatment effects on MVPA outcomes stratified by initial WL (p’s>.65).

## Discussion

This randomized trial investigated the feasibility, acceptability, and preliminary efficacy of a 12-week yoga intervention, delivered following a 3-month behavioral WL program, on changes in body weight and important psychological constructs among women with overweight or obesity. Overall, retention, adherence, and program satisfaction ratings to the yoga intervention were excellent. Treatment groups did not differ on 6-month WL; however, among those who lost ≥5% during the 3-month SBWL treatment, 6-month WL was 3.5% greater in yoga versus control. These data provide preliminary support for the implementation of yoga as a strategy for improving long-term WL among individuals initially achieving a clinically significant WL.

Similar to the pattern observed with WL, the effect of yoga on psychological constructs was predominately seen among those losing ≥5% during SBWL treatment. Specifically, when compared to CON, yoga participants who lost ≥5% exhibited lower negative affect, and greater distress tolerance, overall self-compassion, and mindfulness, as it relates to observing and responding to internal thoughts and feelings in non-judgmental or non-reactive ways. While it is not surprising that yoga influenced these constructs given previous research [19, 21, 24], the fact that this effect was only observed among those with high initial WL is interesting. While we don’t definitively know why this was the case, we hypothesize that success in SBWL treatment (i.e., high initial WL) may have increased WL self-efficacy, self-regulation capacity, and/or belief in the weight-related benefits of yoga, which may not be true for those experiencing lower initial WL. These improved abilities could have created an ‘upward spiral’, generating momentum and enthusiasm as one entered the yoga portion of the program [43]. Consistent with this theory, yoga participants who lost ≥5% initially attended more yoga sessions compared to those with lower initial WL, despite no differences in attendance during SBWL treatment. These data may suggest greater ‘buy in’ regarding the benefits of yoga among those with high initial WL, or alternatively, that the dose of yoga needed to elicit these psychological benefits was not sufficient among those with low initial WL.

The fact that yoga was shown to be superior to control on weight outcomes solely among those who lost ≥5% initially, was not surprising given that the psychological and behavioral processes targeted by yoga more closely resemble the types of skills that correspond to behaviors shown to be predictive of long-term WL success or WL maintenance (e.g., ability to tolerate and cope with stressors, negative mood states, and hedonic urges to eat) [6–8] and not the promotion of initial WL (e.g., self-regulation behaviors such as self-monitoring). As discussed above, increased mindfulness, distress tolerance, and self-compassion, resulting from yoga, could elicit a ‘gateway’ or ‘transfer effect’ on other important weight-control behaviors such as diet or exercise. For example, increased mindfulness could help reduce non-homeostatic eating by increasing awareness to hunger signals or helping to create a pause between having a thought (e.g., that cupcake looks amazing) and acting on that thought (e.g., consuming the food). Similarly, increased distress tolerance could aid in coping with uncomfortable thoughts or feelings that may arise from intense cravings or urges to eat. An increase in self-compassion could help break the vicious cycle that often produces shame following overeating or a dietary lapse, which often leads to all-or-nothing thinking and further poor dietary choices. These examples all point to a ‘transfer effect’, or examples of what yoga practitioners refer to as ‘taking yoga off the mat’. These may be particularly important following the completion of SBWL treatment, when behavioral adherence to dietary regimens is more difficult given the perceived costs of adherence gradually exceed the perceived benefits [4]. Given that formal mediation analyses were unjustified in this study due to limited data points and small sample size, we cannot know for certain whether improvements in these psychological constructs were responsible for group differences in WL among those with high initial WL. Future studies should examine both mediators and moderators to better understand the mechanisms through which yoga exerts an effect on those successful in SBWL treatment.

Several additional findings warrant discussion. First, a common challenge against incorporating yoga into a weight management program is that yoga would be practiced ‘in place of’ more traditional aerobic exercise. Findings from this study refute this claim, indicating that yoga and control participants did not differ in objectively-assessed moderate-to-vigorous intensity physical activity minutes, despite yoga participants engaging in 120 minutes of group-based yoga sessions per week and additional self-initiated yoga practice. Further, it is unlikely that the energy expenditure resulting from the practice of yoga over 12 weeks substantially contributed to WL, given that Iyengar yoga has a relatively low energy expenditure. Second, while it is uncertain why there was a lack of an overall treatment effect of yoga on weight, it appears that this finding was driven by those with low initial WL. Contrary to hypotheses, yoga participants with low initial WL had lower positive affect and higher negative affect at 6 months compared to control participants. It is possible that poor initial WL contributed to a ‘downward spiral’, resulting from lower self-efficacy, and greater frustration due to a lack of perceived benefit of the program, thereby negatively impacting affect, and possibly limiting the effect of yoga on weight. Third, it is interesting to note that all program satisfaction ratings and attendance at yoga classes did not differ between Cohorts 1 and 2, despite Cohort 1 being delivered in-person and Cohort 2 being delivered remotely. In fact, attendance was slightly higher (82% vs. 70%), albeit not statistically significant, for remote-delivered classes. These findings are in line with other remote-based exercise or mind-body interventions which have reported high satisfaction ratings to this remote format [44–46]. They also suggest that remote delivery of yoga may be a potential strategy for improving long-term WL among those with initial WL success, as it offers a more disseminable approach and overcomes many traditional barriers to in-person yoga classes (e.g., geographical constraints, limited time for travel, no childcare). Future research in this area may be critical for improving yoga translation efforts. Finally, many individuals (n = 118) did not want to participate in this study due to the large time commitment required. However, it is unclear whether this number would be reduced if remote-based classes were offered from the beginning (versus in-person classes) or if the yoga or culinary/nutrition education classes were less frequent. This requires further investigation.

This pilot study is strengthened by a novel application of yoga to a weight management program, randomized design, structurally equivalent control condition, and excellent retention, adherence, and program satisfaction ratings. However, it is limited by a relatively small sample size with reduced power, particularly for moderator analyses. Further, the yoga intervention was modified for the second cohort (online vs. in-person, more time in poses, more specific homework instructions), and therefore the effects of these modifications cannot be fully elucidated. Finally, enrollment was limited to women, the majority of whom were non-Hispanic White, thereby limiting the generalizability of study findings to men and to other ethnic and racial groups. Racial and ethnic minorities are greatly under-represented in yoga research and our study sampling methods—which did not explicitly target these subgroups—regrettably contributed to this pattern, while limiting external validity. To address this significant limitation, future work should integrate established recommendations [47–49] for designing recruitment plans that aim to increase diversity within yoga research.

## Conclusion

To our knowledge, this is the first randomized trial to test a yoga intervention following SBWL treatment. Findings indicate excellent feasibility and acceptability. Although there were no differences in WL outcomes between yoga and control, post-hoc analyses suggest that yoga offers promise as potential treatment approach for improving long-term WL among women who achieve a clinically significant WL during SBWL treatment. Future studies, which include men, more individuals from underrepresented racial and ethnic groups, larger sample sizes, and a longer study duration, are needed to confirm these preliminary findings. Further, mechanistic studies which evaluate the psychological and behavioral processes through which yoga may influence body weight are warranted.

## Supporting information

S1 Checklist Consort checklist. (DOC)

S1 File Protocol as approved by the institutional review board. (PDF)
