# Supplementary material for: A preliminary investigation of yoga as an intervention approach for improving long-term weight loss: A randomized trial
Source: PLoS One. 2022 Feb 4;17(2):e0263405. doi: 10.1371/journal.pone.0263405 (PMC8815874; doi:10.1371/journal.pone.0263405)
Supplement: S1 File — (PDF) [file pone.0263405.s002.pdf]

PROTOCOL

Approaches for Improving Long-term Weight loss

Project LEARN Study

5/16/20

NCT03799289

Study Summary. Behavioral weight loss (WL) programs result in clinically significant weight losses; however rates of long-term WL maintenance are poor. Previous studies suggest that long-term WL success may require an enhanced ability to overcome physiological and hedonic urges to eat and an improved capacity for dealing with life stressors, negative mood states, and food cravings. Thus interventions which target stress reduction and reduce the tendency to use food as a coping strategy for aversive experiences may offer a protective effect against dietary lapses; thereby improving long-term WL outcomes. Yoga is a mind-body intervention which reduces stress and improves overall physical and psychological well-being and offers promise for strengthening the psychological skill set needed for maintaining important weight-related behaviors long-term. The physical and cognitive skills practiced within yoga target multiple underlying psychological processes (e.g., mindfulness, distress tolerance) which could reduce emotional eating, improve dietary choices, and enhance one's ability to tolerate food cravings or hedonic urges to eat. While yoga is an effective treatment approach for other chronic health conditions, it has not been examined as a potential intervention for improving long-term WL outcomes. Within the context of the obesity field, yoga has been viewed as a mode of exercise and not necessarily as a mind-body intervention approach (as is the case in other fields). Thus, given the lower caloric expenditure of yoga in comparison to many forms of aerobic exercise, the effect of yoga on important weight-related processes and behaviors has not been examined. This study aims to examine the feasibility and acceptability of implementing yoga within a weight management program and to examine the impact of yoga, relative to a contact-control condition (CON), on important psychological constructs and weight-related behaviors. Sixty women with a BMI of 25-40 kg/m2 will be randomly assigned to a 12-week standard behavioral WL program, followed by either 12 weeks of group-based yoga or 12 weeks of cooking/dietary information classes. Both groups will be instructed to self-monitor and achieve the dietary and aerobic exercise goals throughout the 24-week program. Primary assessments will occur at baseline and weeks 12 and 24. Feasibility and acceptability of the yoga intervention will be assessed as primary aims. Secondary aims include comparing YOGA and CON on general (perceived stress, mindfulness, affect, and distress tolerance) and eating-specific variables (hedonic, emotional, and mindful eating) assessed via questionnaire and via ecological momentary assessment (EMA; dietary lapses and self-control or the ability to resist dietary temptations). Exploratory aims include the comparison of treatment groups on changes in weight and aerobic exercise minutes, as assessed via physical activity monitors. These preliminary findings can be used to inform future research efforts on the efficacy of yoga, an example of a mind-body intervention for WL maintenance, and the psychological mechanisms through which yoga may influence body weight.

Eligibility Criteria

Inclusion criteria:

1) Age 18-60 years
2) BMI between 25.0 and <40.0 kg/m^2
3) Female

Exclusionary criteria include:

1) Presence of any condition that would limit one’s ability to exercise (i.e., orthopedic limitations)
2) Recent weight loss (≥10 pounds within the past 6 months)
3) Current or recent enrollment (<2 year) in a weight loss program or other stress-management or mindfulness-based treatment program (e.g., yoga or Tai)
4) Currently taking any weight loss or other medication that could alter one’s metabolism
5) Women who are pregnant, planning on becoming pregnant in the next 6 months, or those pregnant within the past 6 months
6) Uncontrolled hypertension (currently taking blood pressure medication or having a resting systolic blood pressure ≥140/90 mmHg)
7) Serious psychiatric disorder (e.g., psychosis, major depression, suicidality)
8) Cognitive or physical limitations that could preclude the use of a smart phone device.
9) Participants with a history of diabetes or heart disease will be considered for this study only if their heart disease or diabetes is well controlled, and physician consent will be required.

Recruitment. A total of 60 participants will be recruited through advertisements in newspapers, the Internet, and on our study center website. Participants viewing Internet ads will have the opportunity to complete a Pre-Screen Survey to assess for initial eligibility by clicking on a link that will bring them to Lifespan's secure REDCap site. After reviewing the responses to the Pre-Screen Survey, eligible participants will then be screened via telephone, along with all other interested participants, to determine eligibility.

Overall study design. Participants will be randomized at baseline to either standard behavioral WL (SBWL) followed by yoga (YOGA) or SBWL followed by a contact-matched control (CON). Randomization will occur at baseline, rather than after 12 weeks of SBWL, so that participants can remain with the same group of peers throughout the entire intervention period. However the participants and therapist will not be informed of their randomization assignment until the initial 12-week SBWL program is complete. Participants in both treatment groups will receive a 12-week, group-based SBWL treatment program, with weekly visits, followed by either 12 weeks of yoga (2x/week) or 12 weeks of cooking/dietary education classes (2x/week) to match for contact time. Assessments will occur at baseline, post-SBWL (12 weeks), post-intervention (24 weeks), and following a 6-month no-contact follow-up period (52 weeks).

SBWL intervention common to both treatment groups: The 12-week SBWL program is modeled after the Diabetes Prevention Program and Look AHEAD trial and is designed to produce a 1-2 lb WL per week. Participants will be given a calorie intake goal of 1200-1800 kcals/day and will be instructed to increase moderate-to-vigorous intensity PA. Participants will start with a goal of 75 min/week and will be encouraged to strive towards the final goal of 200 min/week of PA. All participants will attend weekly, group-based sessions, and will be taught key behavioral strategies to assist in modifying dietary and PA behaviors (e.g., stimulus control, problem-solving, goal-setting), and classes will be held separately for YOGA and CON. Participants will also be instructed to weigh themselves daily and to self-monitor dietary intake and PA. Following the 12-week program, participants will be encouraged to continue on a reduced calorie diet, to engage in 200 min/wk of aerobic PA (independent of yoga practice), and to continue to self-monitor dietary intake, weight, and PA.

Yoga intervention: Participants assigned to YOGA will receive a 12-week, group-based yoga intervention following the 12-week SBWL program. Participants will attend two, 60-minute Iyengar yoga sessions per week at the WCDRC, the same location as the SBWL program. Iyengar yoga, is a form of hatha yoga, which incorporates breathing, postural, and mediation practices and focuses on the precision in movement and attention to subtle aspects of posture and breath, as a means of developing improved self-awareness and mindfulness (i.e., staying in the present moment). A hallmark of Iygengar yoga is the utilization of 'props' (e.g., straps, blocks, blankets), to assist individuals in maintaining the proper alignment and to reduce the risk of injury. Further, Iyengar yoga was also chosen because it is less aerobic (i.e., 'light' intensity PA) than other forms of yoga (e.g., power yoga), placing more emphasis on meditation, breath control and correct postural alignment, and thus helps to avoid a confound with assessments of aerobic PA. Bi-weekly group classes will be led by a certified yoga instructor. Classes will consist of breathing exercises, seated postures, standing postures, brief guided meditation, directing participants to focus on breath and body awareness. Participants will also be given notecards which encourage participants to apply the cognitive skills practiced within class (e.g., mindfulness, and distress tolerance) to personal experiences or struggles which they may encounter outside of class, with specific reference to adherence to weight control prescriptions. Sequences will be finalized with the input of the yoga instructor and investigators, and participants will be encouraged to practice yoga at home. To help facilitate home-based yoga practice, participants will be provided printed handouts and audio/video recordings. Note: due to COVID-19, the last 10 weeks of Cohort 2 yoga sessions were conducted remotely, via live Zoom sessions vs. in-person classes.

Cooking & dietary information intervention: Participants randomized to CON will receive 12 weeks of cooking and dietary information classes following the 12-week SBWL program. Similar to those randomized to the yoga intervention, CON participants will participate in 2 weekly group sessions led by a culinary expert from Johnson & Wales University. These classes will include cooking demonstrations, food taste testing, and basic cooking/dietary knowledge. We chose a cooking/dietary information intervention as the comparison condition to yoga for several reasons: 1) we felt that it was important to match for contact time to control for the potential effect of group or staff support on outcome measures, 2) we felt that it was important to provide participants with a relevant intervention in order to promote attendance at group sessions (versus a more true 'control' condition, such as general wellness videos), 3) we wanted a comparison condition that would likely not

influence many of the key psychological constructs impacted by yoga (e.g., mindfulness, stress reduction, distress tolerance), 4) we did not want to compare yoga to aerobic exercise before first showing the initial benefits of yoga on psychological constructs and weight-related behaviors, and 5) we didn't want a comparison condition which taught behavioral strategies or skills, rather one which focused more on general education. Note: due to COVID-19, the last 10 weeks of Cohort 2 cooking/dietary information sessions were conducted remotely, via live Zoom sessions vs. in-person classes.

Assessments: Assessments will occur at baseline, 12 weeks, 24 weeks, and 52 weeks. A demographics questionnaire will be completed at baseline and all remaining questionnaires will be assessed at all 4 time points.

Relevant psychological constructs: Several psychological constructs, which have been shown to be impacted by yoga will be assessed via previously validated questionnaires. Perceived stress will be measured via the Perceived Stress Scale, which assesses the degree to which situations in one's life are appraised as stressful. Dispositional mindfulness will be assessed via the Five Facet Mindfulness Questionnaire. This questionnaire assesses one's ability to observe, describe, and act with awareness, and be non-judgmental and non-reactive to one's inner experience. Affect will be assessed via the Positive and Negative Affect Schedule which consists of two, 10-item scales to measure both positive and negative affect. Distress tolerance will be measured via the Distress Tolerance Scale and the Discomfort Intolerance scale, which measures one's perceived capacity to tolerate distress. Self-compassion will be assessed using the Self-Compassion Scale which assesses the degree to which an individuals acts twards themselves when experiencing a difficult time.

Eating-specific constructs: Emotional eating will be measured via the Emotional Eating Scale which examines the extent to which certain feelings lead to the urge to eat (e.g., eating in response to anger, anxiety, and depression). Hedonic eating refers to the drive to eat or consumption of food for pleasure, in the absence of an energy deficit. The Power of Food Scale will be used to assess hedonic hunger as it measures appetite for palatable foods at three levels of food proximity (food available, food present, food tasted). Mindful eating refers to a non-judgmental awareness of physical and emotional sensations associated with eating and will be assessed via the Mindful Eating Questionnaire which has five factors (disinhibition, awareness, external cues, emotional response, and distraction). Binge eating will be assessed via the Binge Eating Scale, which measures the emotional/cognitive symptoms associated with binge eating, and via EMA (see below). Restrained and disinhibited eating will be assessed using the Three-Factor Eating Quesitonnaire which meaures three dimensions of eating behavior.

Weight-related constructs: Weight bias and weight stigma will be assessed using the Weight Bias Internalization Scale and Weight Stigma Questionnaire respectively. Important weight-control behaviors will be assessed using the Weight Control Strategies Questionnaire which consists of 4 subscales: dietary choices, self-monitoring strategies, physical activity, and psychological coping.

EMA methodology: Participants will carry a smartphone for 10 days at baseline, 12, and 24 weeks. In most instances this will be their own personal phone. However, in the case that a participant is unable to receive text messages using our automated system, a phone will be provided to the participant to be used only during the 10-day assessment period (i.e., will be returned to research staff immediately following this period). Participants will respond to text message surveys and will report on mood and hunger levels, dietary temptations, food cravings, and dietary lapses as they occur in their natural environment. All entries will be made in response to 5 semi-random prompts delivered throughout the day via an audible tone. Upon hearing the signal, participants will be instructed to answer questions via the smartphone within 45 minutes. Each survey will take 2-5 minutes to complete and individuals <80% compliant at baseline will be ineligible.

Participants will be asked whether they experienced any food cravings (i.e., intense desire to eat a certain food) and dietary temptations (i.e., a sudden urge to overeat, or eat a forbidden food, which could be due to internal or external factors) since the last prompt. If the response is 'yes', participants will rate the intensity of the craving, difficulty in resisting the food, anticipated pleasure if they eat the food, and whether the food was eaten. If the food was consumed, they will indicate how they felt after eating the food. Tolerance for resisting dietary temptations will be defined as the percentage of instances in which a dietary temptation or food craving

was present, but the food was *not* consumed. Given that dietary lapses may also occur in the absence of food cravings or dietary temptations, participants will also indicate whether they had eaten since the last prompt. If 'yes', they will be queried about this eating episode. A dietary lapse will be defined as 'eating past the point of feeling full', 'eating more than usual', 'unplanned eating' (i.e., consuming food when I don't usually eat and not making up for a missed meal), or 'eating a food not appropriate for their plan'. A binge eating episode will be defined as any eating episode in which a participant reports eating an 'unusually large amount of food' while also reporting a loss of control during eating (e.g., inability to stop eating). Data on stress, mood, hunger, and environmental stimuli will also be collected at each prompt. This will allow for the examination of whether particular factors preceded dietary lapses or predicted tolerance for food cravings or dietary temptations.

Exploratory measures: Body weight will be measured at each assessment visit using standard procedures. Physical activity will be objectively-assessed via the previously-validated Sensewear armband (Body Media Inc.) and participants will wear the armband for 10 consecutive days (to coincide with each EMA monitoring period). Total min/wk spent engaging in moderate-to-vigorous intensity PA (MVPA; ≥3 metabolic equivalents) and bout-related MVPA (≥10 min bouts; a measure of structured exercise) will be calculated.

Acceptability and feasibility measures: We will compare YOGA and CON on adherence to the intervention and retention at the 24-week assessment to ensure feasibility of YOGA relative to a contact-matched control. Acceptability will be assessed by a program satisfaction questionnaire.

Participant Compensation. Participants will receive $25 for the completion of the 12-, 24-, and 52-week assessment visits and will have the opportunity to earn an additional $50 at the 12- and 24- week assessments for completion of EMA procedures ($0.50 per survey completed and a $25 bonus if response rate is >85%).
